# A synthetic *Bacillus* compound agent enhances cotton yield and fiber quality by regulating rhizosphere microbes and metabolites

**DOI:** 10.3389/fpls.2026.1774588

**Published:** 2026-03-09

**Authors:** Qingpeng Wang, Chengcai Yan, Qingyun Liu, Wenqing Gao, Lan Wang, Hongqiang Dong, Guodong Chen, Haiting Hao

**Affiliations:** 1 Key Laboratory of Integrated Pest Management in Southern Xinjiang, Alar, China; 2 Scientific Observing and Experimental Station of Crop Pests in Alar, Ministry of Agriculture, College of Agriculture, Tarim University, Alar, China; 3 Key Laboratory of Genetic Improvement and Efficient Production for Specialty Crops in Arid Southern Xinjiang of Xinjiang Corps, Tarim University, Alar, China

**Keywords:** cotton, fiber quality, rhizosphere soil microbial community, rhizosphere soil secondary metabolites, synthetic bacillus compound agent, yield

## Abstract

To improve cotton yield and reduce reliance on chemical fertilizers and pesticides, we synthesized a novel compound microbial agent with cotton growth-promoting activity. Pot experiments demonstrated that application of this agent significantly promoted cotton seedling growth: chlorophyll content increased by 6.6%, root activity was enhanced by 87.69%, and malondialdehyde (MDA) content decreased by 44.36%. Furthermore, the agent treatment significantly enhanced the antioxidant enzyme activities in cotton seedlings, with catalase (CAT), peroxidase (POD), and superoxide dismutase (SOD) activities increasing by 136.56%, 125.00%, and 45.73%, respectively. Field plot trials showed that the agent increased cotton yield by 20.00%–24.34% and improved fiber quality, with the spinning consistency index, breaking fiber strength, and elongation increased by 7.35%, 7.48%, and 3.06%, respectively. Further analyses of nutrient contents in cotton leaves, rhizosphere soil physicochemical properties, amplicon sequencing, and untargeted metabolomics revealed that, compared with the control group: nitrogen (N), phosphorus (P), and potassium (K) contents in cotton leaves increased by 33.27%, 68.24%, and 37.76%, respectively; rhizosphere soil pH decreased by 2.78%, while available phosphorus (AP), organic matter (OM), and total phosphorus (TP) increased by 33.43%, 17.40%, and 73.26%, respectively; bacterial community Chao1 index decreased by 7.93%, whereas the Shannon index increased by 0.29%; fungal community Chao1 and Shannon indices increased by 17.54% and 1.07%, respectively. At the genus level, the relative abundances of *Iamia*, *Polycyclovorans*, *Arenimonas*, and *Verticillium* increased by 43.48%, 10.51%, 31.27%, and 67.58%, respectively, whereas those of *Bacillus* and *Fusarium* decreased by 35.37% and 21.00%, respectively. Moreover, the secondary metabolite composition in rhizosphere soil was altered: terpenoid and nitrogen-containing compound contents increased, while phenol content decreased, with significant accumulation of tryptamine, L-tryptophan, and serotonin. This study confirms that the novel compound microbial agent enhances cotton yield and fiber quality via synergistic mechanisms, providing effective technical support for the development of green cotton production.

## 1 Introduction

Cotton, a globally dominant fiber and oilseed crop, is critical to the textile, energy, and chemical industries, serving as both a commercial commodity and a strategic material (Jans et al., 2021). As the world’s largest producer, China contributes a quarter of the global supply, with an annual output of approximately 6 million tons, and depends heavily on Xinjiang’s unique agroclimatic conditions and advanced agronomic practices (Li et al., 2020; Feng et al., 2024). Central to this output is Xinjiang, a region in northwestern China that has evolved into the nation’s agricultural linchpin. It contributes over 90% of China’s total cotton production (more than 20% globally), cultivates 2.5 million hectares of high-quality cotton fields (82.8% of the national total), and involves 50% of local farmers (Zhou et al., 2024b). Designated as a core “platinum industry,” it is a strategic pillar for the national cotton supply chain and a cornerstone of rural revitalization, providing stable support for agricultural economic security (Wang et al., 2021). However, despite its paramount socioeconomic significance, Xinjiang’s cotton production faces several pressing sustainability challenges.

Recent adjustments in national agricultural policies have encouraged a partial shift in Xinjiang’s cotton farmland toward staple crops such as wheat and maize, leading to reduced cotton cultivation area and heightened pressure on textile raw material supply (Zhao et al., 2025b). Meanwhile, the sustainability of remaining cotton production is severely threatened by continuous cropping obstacles: long-term monocropping accumulates autotoxic root exudates (e.g., free amino acids and phenolic compounds), which degrade the rhizosphere environment, disrupt soil microbial community structure, reduce microbial diversity, and promote soil-borne pathogens (e.g., *Fusarium oxysporum*, *Verticillium dahliae*), ultimately increasing the incidence of blight and *Verticillium* wilt and causing yield and quality losses (Feng et al., 2020; Xu et al., 2021; Zhang, 2021; Ma et al., 2023).

To address these challenges, conventional management primarily relies on heavy chemical fertilizers, pesticides, and resistant cultivar breeding (Gürsoy, 2024). However, this approach involves critical trade-offs: prolonged agrochemical overuse induces soil microbial dysbiosis and organic matter depletion (Wang et al., 2022; Xiao et al., 2024), while breeding strategies are constrained by long development cycles and limited genetic diversity—particularly for intractable soil-borne diseases like cotton *Verticillium* wilt (Zhang et al., 2025b). Consequently, microbial regulation technologies have emerged as a sustainable alternative, as they reconstruct healthy rhizosphere microecology and improve soil physicochemical properties to alleviate continuous cropping obstacles and boost yield (Chen et al., 2024; Gao et al., 2024; Li et al., 2024; De freitas et al., 2026).

As environmentally friendly biocontrol and growth-promoting agents (microbial agent) (El-Saadony et al., 2022), microbial agents have attracted growing attention in the global agricultural sector over recent years (El-Saadony et al., 2022). These agents refer to preparations composed of beneficial microorganisms (or their metabolites) that can suppress plant pathogens, promote crop growth, and improve soil fertility without residual pollution. Studies have demonstrated that the application of single-strain or compound microbial agents can significantly reduce plant disease incidence, improve soil quality, and promote crop growth (Vitale et al., 2024). Compared with traditional chemical pesticides and fertilizers, microbial agents offer distinct advantages such as ecological sustainability and zero residual pollution, thereby positioning them as critical technological tools for green agricultural development. In cotton cultivation, the application of single agents has achieved notable progress. For instance, *Bacillus subtilis* exhibits antagonistic activity against *Verticillium dahliae*, thereby significantly reducing the incidence of cotton *Verticillium* wilt (Zhao et al., 2022). Nitrogen-fixing bacteria and phosphorus-solubilizing bacteria enhance the utilization efficiency of soil nutrients (e.g., nitrogen and phosphorus), thereby promoting root development in cotton (Zhou et al., 2024a; Zhao et al., 2025a). However, single microbial agents inherently exhibit functional singularity and poor environmental stability: their field efficacy is highly sensitive to soil physicochemical properties, climatic conditions, and other environmental factors, leading to unstable and inconsistent application effects in practical production. This shortcoming is particularly prominent in Xinjiang’s arid and variable agroecological environment. In contrast, compound microbial agents integrate multiple functions (e.g., disease suppression, growth promotion, and stress tolerance) via synergistic interactions among different microbial strains, as evidenced by studies in other crops showing that co-application of different beneficial microbes leads to superior outcomes compared to single-strain applications (Peng et al., 2023). Given the unique challenges of continuous cropping obstacles and agrochemical overuse in Xinjiang’s cotton production, systematic research on the application of compound microbial agents is lacking, creating an urgent need to explore feasible biological solutions.

To develop efficient, stable growth-promoting and disease-suppressing microbial agents for cotton, our research group previously screened strains with excellent biocontrol activity, strong growth potential, and environmental adaptability from saline-alkali land and medicinal plants, and established a high-quality strain library (Dai, 2024; Zu, 2024). From this library, four *Bacillus amyloliquefaciens* strains (DT2-1, MR4, KL9, KS7) were selected to synthesize a compound microbial agent. Pot experiments were conducted to evaluate the efficacy of the agent in promoting cotton seedling growth, in comparison with single-strain and commercial microbial agents. Field trials compared the compound and single-strain agents for their effects on soil properties, rhizosphere microbial communities, soil metabolite profiles, and cotton yield and fiber quality. The objectives of this study were to clarify the efficacy of the synthesized compound microbial agent in promoting cotton seedling growth and its superiority over single-strain and commercial agents, the mechanisms by which the compound agent improves soil physicochemical properties, reshapes rhizosphere microbial communities, and regulates soil metabolite profiles, and the relationship between compound agent-induced changes in rhizosphere microecology and improvements in cotton yield and fiber quality. The findings aim to provide a feasible biological strategy for alleviating continuous cropping obstacles in Xinjiang cotton production, reducing reliance on chemical fertilizers and pesticides, and advancing sustainable agricultural practices.

## 2 Materials and methods

### 2.1 Experimental material

The cotton cultivar used in this study was ‘Tahe 2’, a major cultivar cultivated in southern Xinjiang, provided by Xinjiang Tarim River Seed Industry Co., Ltd. Five microbial agents were tested in this research, comprising two synthetic formulations and three commercially available products: (1) synthetic *Bacillus* compound agent (C): Mixed four *Bacillus amyloliquefaciens* strains (DT2–1 from *Codonopsis pilosula* rhizosphere soil, MR4 from *Oxytropis miliacea* endophytes, KL9 and KS7 from *Allium platyspathum* endophytes) at a 1:1:1:1 ratio for fermentation. After adding freeze-drying protectant, vacuum freeze-drying, and carrier adsorption, the final viable bacterial count was ≥ 3.5 × 10^10^ CFU/g; (2) synthetic single *Bacillus* agent (S): Prepared using strain DT2–1 through the same process as the compound agent, with a viable bacterial count ≥ 3.0 × 10^10^ CFU/g; (3) Commercial *Trichoderma harzianum* agent (H): Viable fungal count ≥ 1.0 × 10^9^ CFU/g, registered under Microbial Fertilizer Registration Certificate No. (2018) Zhunzi (3447), provided by Hubei Qiming Bioengineering Co., Ltd.; (4) Commercial *Bacillus subtilis* agent (K): Viable bacterial count ≥ 2.0 × 10^8^ CFU/g, registered under Microbial Fertilizer Registration Certificate No. (2020) Zhunzi (8062), provided by Henan Shuanghui Agricultural Science and Technology Development Co., Ltd.; (5) Commercial *Bacillus* compound agent (F): Viable bacterial count ≥ 2.0 × 10^9^ CFU/g, registered under Microbial Fertilizer Registration Certificate No. (2022) Zhunzi (11441), provided by Beihai Yiqiang Biotechnology Co., Ltd.

### 2.2 Cotton seedling experiment

A greenhouse pot experiment was conducted to evaluate the effects of different microbial agents on cotton seedling growth and stress resistance. Six treatments were set up: synthetic *Bacillus* compound agent (C), synthetic single *Bacillus* agent (S), commercial *Bacillus* compound agent (F), commercial *Bacillus subtilis* agent (K), commercial *Trichoderma harzianum* agent (H), and sterile water control (W), with nine biological replicates per treatment.

Cotton seeds were surface-sterilized with 75% ethanol for 3 minutes, then germinated in darkness at 30 °C for 3–5 days. Uniform seedlings were transplanted into plastic pots (10.0 cm × 10.0 cm × 9.0 cm) filled with 150 g of sterilized substrate (peat: vermiculite = 3:1, v/v), with two seedlings per pot. At the two-leaf one-terminal-bud stage (7 days after transplantation), each pot was treated with 50 mL of microbial agent suspension (5.25 × 10^7^ CFU/mL) via root drenching, while the control group (W) received an equal volume of sterile water. Cotton seedlings were harvested 45 days after treatment to determine growth and physiological indicators related to stress resistance: growth indicators included plant height, stem base diameter, root length, shoot fresh weight, root fresh weight, shoot dry weight, and root dry weight; physiological indicators included chlorophyll content, activities of antioxidant enzymes [catalase (CAT), peroxidase (POD), superoxide dismutase (SOD)], root activity, and malondialdehyde (MDA) content (a marker of membrane lipid peroxidation).

### 2.3 Determination of growth and physiological parameters

To ensure the accuracy and standardization of the test results, the following standardized methods were adopted for the determination of growth and physiological parameters of harvested cotton seedlings. Plants were carefully uprooted and rinsed with deionized water to remove adhering soil. After air-drying, plant height and root length were measured using a standard ruler, and stem basal diameter was determined with a digital vernier caliper. For biomass measurement, twelve seedlings per treatment were selected for fresh weight determination using an analytical balance. The samples were then oven-dried at 105 °C for 30 min, followed by drying at 80 °C to constant weight for dry weight recording.

Physiological and biochemical analyses were performed as follows: (1) Chlorophyll content was determined spectrophotometrically after extracting 0.5 g of fresh leaf tissue (from the second fully expanded leaf) with 95% ethanol. (2) Antioxidant enzyme activities and malondialdehyde (MDA) content were analyzed using commercial assay kits. Catalase (CAT) activity was measured by the ultraviolet colorimetric method, superoxide dismutase (SOD) and peroxidase (POD) activities were determined by colorimetric methods, and MDA content was quantified using the thiobarbituric acid method. (3) Root vitality was assessed using the triphenyl tetrazolium chloride (TTC) method (Ali et al., 2021).

### 2.4 Cotton field experiment

The field trial was conducted in a 3-hectare sandy loam cotton field in Chang’an Town, Alar City, Xinjiang (40°34′57″N, 81°19′16″E), which features a warm-temperate arid desert climate (annual precipitation: 72 mm, mean annual temperature: 11.4 °C, abundant sunshine, high evaporation) (Zhu et al., 2023). The cotton cultivar ‘Tahe 2’ was sown on May 10, 2024, using a plastic film-mulched drip irrigation system (one film, two drip tapes, six rows per film) with a planting configuration of (60 + 10) cm row spacing, 10 cm plant spacing, and 3–4 cm sowing depth.

A completely randomized block design was adopted, with two zones selected based on soil fertility and moisture gradients: central zone (fertile, adequate moisture) and edge zone (infertile, water-deficient). Each zone (1,800 m²) was divided into three 600 m² treatment subplots, with the following treatments: (1) *Bacillus* compound microbial agent (C1: central zone, C2: edge zone); (2) *Bacillus* single agent (S1: central zone, S2: edge zone); (3) Sterile water control (CK1: central zone, CK2: edge zone). Root drenching was performed twice (seedling and squaring stages) with 50mL per plant of microbial agent solution (5.25×10^7^ CFU/mL); control groups received an equal volume of sterile water. All plots were managed uniformly. Subsequent investigations and determinations were conducted at the appropriate growth stages, including analyses of cotton rhizosphere soil microbial community, untargeted metabolites, and soil physicochemical properties, as well as the determination of leaf nutrient elements; additionally, field disease control efficacy, cotton yield, and fiber quality will be systematically investigated and measured to comprehensively evaluate the application effect of microbial agents.

### 2.5 Determination of leaf nutrient elements and soil physicochemical properties

On August 31, 2024, leaf and rhizosphere soil samples were collected from each treatment plot. For leaf samples: ten uniformly growing cotton plants were randomly selected per plot, and the 3rd-4th fully expanded functional leaves from the upper-middle canopy were collected, sealed in sampling bags, and transported on dry ice to Nanjing Aoqing Biotechnology Co., Ltd. for analysis. Leaf nitrogen (N), phosphorus (P), and potassium (K) contents were determined following the National Standard (GB 5009.268-2016) with three biological replicates.

For rhizosphere soil samples: collected via the root-shaking method, sieved through a 2mm mesh, and homogenized. A portion of each sample was air-dried for physicochemical analysis: soil pH was measured with a digital pH meter (soil-to-water ratio = 1:2.5, three technical replicates); other parameters (available K, available P, available N, organic matter, total N, total P, total K) were analyzed by Nanjing Aoqing Biotechnology Co., Ltd. According to national/industry standards with six biological replicates.

### 2.6 Soil microbial community analysis

Rhizosphere soil samples were collected and homogenized as described in Section 2.5, then immediately transferred to sterile cryotubes, flash-frozen in liquid nitrogen, and transported on dry ice to Novogene Co., Ltd. for amplicon sequencing. Total microbial DNA was extracted via the CTAB method. After quality verification, PCR amplification was performed to target the bacterial 16S rRNA V3–V4 region (primers: 341F/806R) and the fungal ITS1-5F region (primers: 1737F/2043R). Amplicons were purified and sequenced on the Illumina NovaSeq 6000 platform (paired-end 250 bp, PE250). Raw data were processed using QIIME2: DADA2 was used to denoise and generate amplicon sequence variants (ASVs); bacterial and fungal ASVs were taxonomically annotated against the Silva 138.1 and UNITE v9.0 databases, respectively. α-diversity (Shannon, Chao1) and β-diversity (Bray–Curtis) indices were analyzed. All analyses of microbial diversity included three biological replicates per treatment, with sequencing depth standardized via rarefaction prior to data analysis.

### 2.7 Untargeted metabolomic analysis

Rhizosphere soil samples for untargeted metabolomics were collected as described in Section 2.5. After homogenization, the soil samples were immediately placed in sterile cryotubes, transported on dry ice to Novogene Co., Ltd., and then analyzed, with three biological replicates per treatment. Pretreatment of each soil sample included: weighing 1.0g of soil; adding 1,000μL of pre-cooled 80% methanol; vortexing and incubating on ice; and centrifuging at 15,000×g and 4 °C for 20 minutes. The supernatant was freeze-dried, filtered through a 0.22μm filter membrane, reconstituted in 200μL of methanol, and spiked with internal standards prior to analysis.

Chromatographic separation was performed using a Hypesil Gold column (column temperature: 40 °C; flow rate: 0.3mL/min) with an acetonitrile gradient. Mass spectrometry was performed on a Q Exactive HF-X Orbitrap in both positive and negative ion modes, with resolutions set at 120,000 (Full MS) and 30,000 (MS²). Raw data were processed using XCMS software for peak extraction and normalization. Metabolites were identified via matching to Novogene’s in-house database, the Human Metabolome Database (HMDB), and the Kyoto Encyclopedia of Genes and Genomes (KEGG). Differential metabolites were screened using multivariate and univariate analyses, followed by KEGG pathway enrichment analysis. Quality control samples were analyzed to ensure the stability of the analytical system and the reliability of the data.

### 2.8 Determination of cotton yield and fiber quality

On September 18, 2024 (cotton harvest stage), the five-point sampling method was adopted to determine the effects of different microbial treatments on cotton yield and fiber quality. Five sampling points were set in each treatment plot, with 50 normally growing cotton plants continuously selected at each point, totaling 250 plants per plot for subsequent determination of yield and quality indicators. The collected cotton plants were subjected to seed removal treatment, and the total weight of seed cotton and lint cotton was weighed and recorded separately. The lint percentage was calculated according to the formula (lint weight/seed cotton weight × 100%). Fiber quality analysis was conducted by the Cotton Quality Supervision, Inspection and Testing Center (Ministry of Agriculture and Rural Affairs, Anyang, China) with a USTER HVI 1000 tester. Measured parameters included upper half mean length uniformity index, fiber strength, micronaire, elongation, reflectance, yellowness, and spinning consistency index. All determinations complied with ISO 2403:2012, with three technical replicates for each sample.

### 2.9 Statistical analysis

All experimental data were first collated and organized using Microsoft Excel 2021. SPSS 26.0 software was employed to perform one-way analysis of variance (ANOVA), followed by the least significant difference (LSD) test to identify significant differences among different treatments, with the significance level set at *P < 0.05.* All data are expressed as mean ± standard deviation (SD). All charts and graphs were generated using Origin 2021 and further optimized with Adobe Illustrator 2021; lowercase letters in the figures indicate significant differences among treatments.

## 3 Results

### 3.1 Effects of microbial agent on cotton growth and physiology

**Figure 1:**
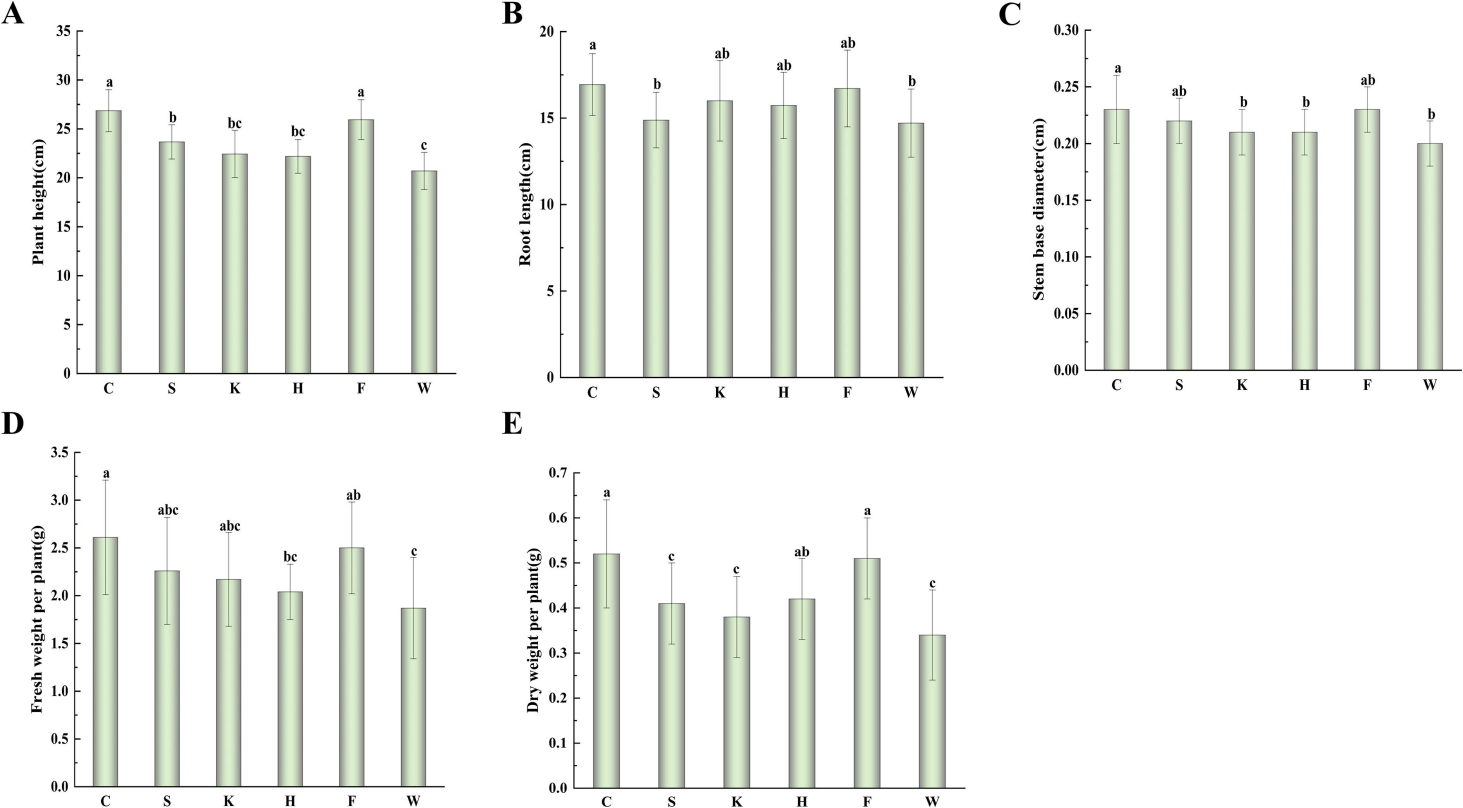
Effects of microbial agent treatments on cotton seedling growth. **(A)** plant height; **(B)** root length; **(C)** stem base diameter; **(D)** fresh weight per plant; **(E)** dry weight per plant. Different lowercase letters indicate significant differences among treatments (*P < 0.05*).

For growth traits (**Figure 1**) C treatment again showed the most pronounced promotion effects relative to W: plant height increased by 29.70%, root length by 15.16%, stem base diameter by 15.00%, fresh weight per plant by 39.57%, and dry weight per plant by 52.94%. Treatment F yielded similar trends (plant height: 25.25%; dry weight: 50.00%), whereas all single-agent treatments produced weaker effects. Notably, treatment C increased dry weight by 26.83% compared with its corresponding single agent S, confirming that inter-strain synergies in compound formulations enhance growth-promoting efficacy. Greenhouse pot experiments demonstrated that microbial agents significantly influenced cotton seedling physiology and growth, with compound agent (synthetic C and commercial F) outperforming single agent (S, K, H) and the sterile water control (W), and C exhibiting the strongest overall efficacy.

**Figure 2:**
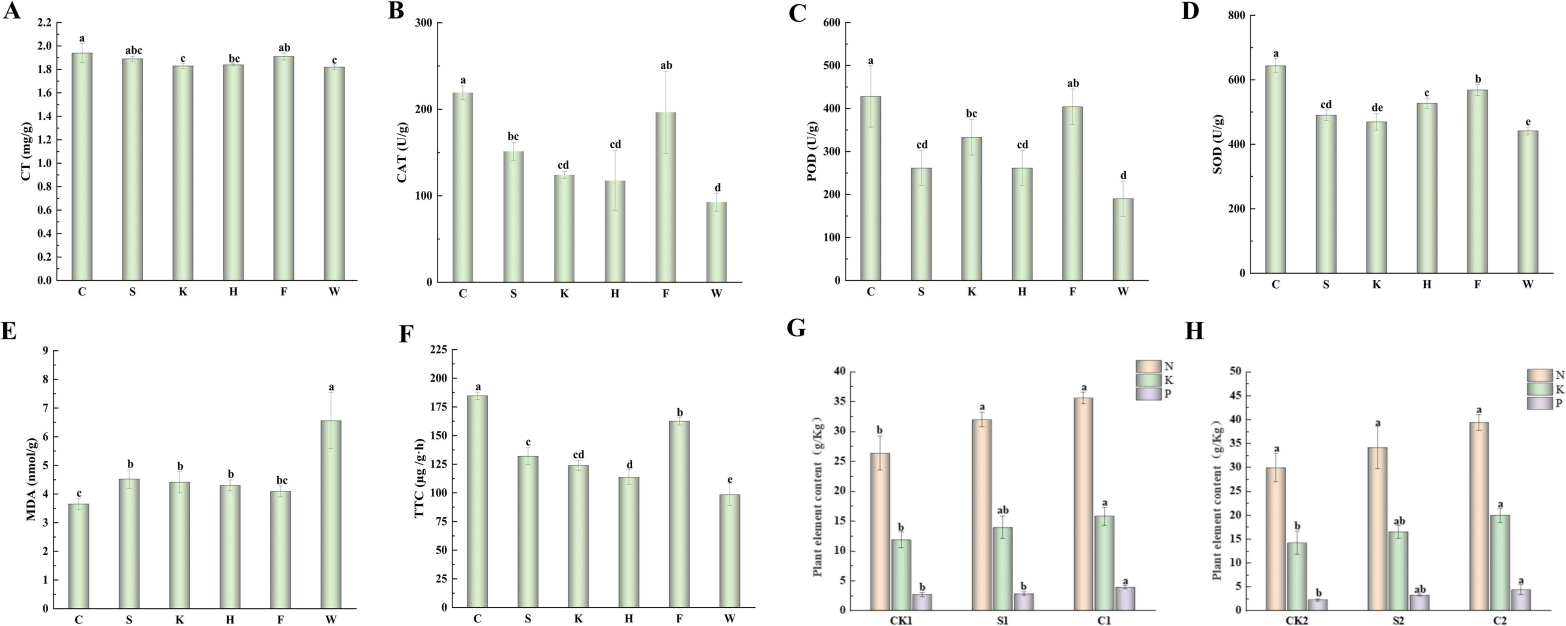
Effects of microbial agent treatments on cotton physiological and biochemical indices. **(A)** total chlorophyll content; **(B)** catalase; **(C)** peroxidase; **(D)** superoxide dismutase; **(E)** malondialdehyde; **(F)** triphenyl tetrazolium chloride; **(G)** Effects on nitrogen (N), phosphorus (P), and potassium (K) contents in cotton leaves from Plot 1; **(H)** Effects on nitrogen (N), phosphorus (P), and potassium (K) contents in cotton leaves from Plot 2. Different lowercase letters indicate significant differences among treatments (*P < 0.05*).

For physiological indicators (**Figure 2**), C treatment significantly enhanced stress resistance-related traits compared to W: chlorophyll content increased by 6.59% (improving photosynthetic capacity), antioxidant enzyme activities were elevated (CAT: 136.56%, POD: 124.91%, SOD: 45.73%), and root activity (TTC reduction) increased by 87.69% (enhancing nutrient uptake). Meanwhile, MDA content (a marker of membrane lipid peroxidation) decreased by 44.36%, indicating alleviated oxidative damage. Furthermore, compound agents showed clear synergistic advantages over single agents: compared to synthetic single agent S, C increased CAT by 44.77%, POD by 63.64%, and root activity by 39.95%; compared to commercial single agents K and H, F increased key enzyme activities and root activity by over 20%. In parallel field trials, microbial agents also promoted cotton leaf nutrient accumulation. In Plot 1, C1 increased leaf nitrogen, potassium, and phosphorus by 35.07%, 33.53%, and 46.79% relative to CK1; in Plot 2, C2 further elevated these nutrients by 31.69%, 41.29%, and 93.95% (phosphorus with the largest increase). Compared with single agents, C1 and C2 increased phosphorus content by 38.43% and 34.35% relative to S1 and S2, respectively, revealing that compound agents boost nutrient uptake via microbial synergism, especially in phosphorus activation.

### 3.2 Effects of microbial agents on soil nutrients, disease control efficacy and rhizosphere microbial community structure

**Figure 3:**
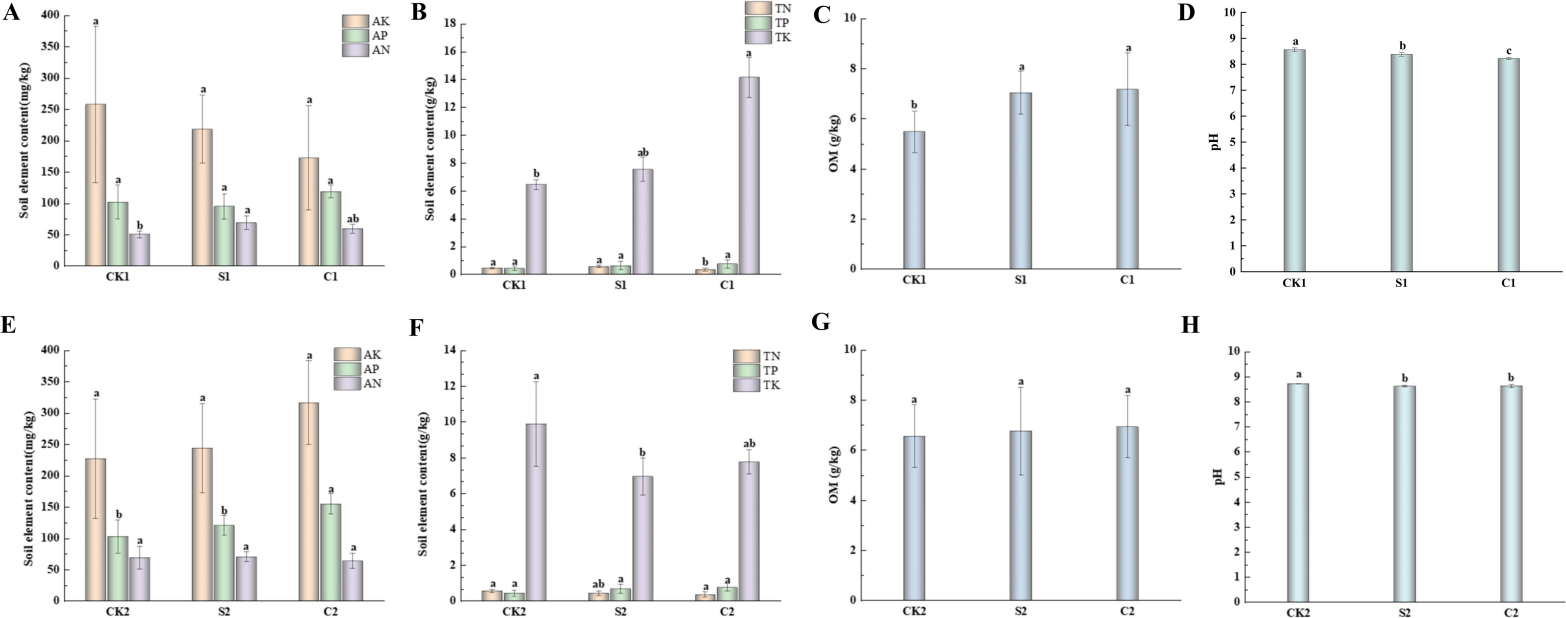
Effects of microbial agents on cotton field soil nutrients. **(A–D)** Impacts on soil elemental contents in Plot 1: **(A)** Available nitrogen (AN), phosphorus (AP), and potassium (AK); **(B)** Total nitrogen (TN), phosphorus (TP), and potassium (TK); **(C)** Organic matter (OM); **(D)** pH value. **(E–H)** Impacts on soil elemental contents in Plot 2: **(E)** Available nitrogen (AN), phosphorus (AP), and potassium (AK); **(F)** Total nitrogen (TN), phosphorus (TP), and potassium (TK); **(G)** Organic matter (OM); **(H)** pH value. Different lowercase letters indicate significant differences among treatments (*P < 0.05*).

Based on the positive outcomes of greenhouse pot experiments, field trials were conducted to systematically investigate the impacts of microbial agents on cotton rhizosphere soil nutrient status, *Fusarium* and *Verticillium* wilt control efficacy, and rhizosphere microbial community assembly. Firstly, rhizosphere soil nutrient dynamics were analyzed (**Figure 3**). In Plot 1, compared with the CK1, the compound microbial agent C1 significantly increased available phosphorus, total phosphorus and total potassium by 16.53%, 76.19%, and 119.41%, respectively. In Plot 2, C2 further elevated AP, TP, and available potassium by 50.18%, 70.45%, and 39.60%, respectively. Compound agents exhibited superior performance over single agents in activating soil phosphorus and potassium pools: in Plot 1, TK content in C1 treated soil was 87.90% higher than that in S1 treated soil; in Plot 2, AP and AK contents in C2 treated soil were 28.43% and 29.87% higher than those in S2 treated soil, respectively. Notably, compound agents displayed robust environmental adaptability, maintaining stable nutrient activation efficacy across different soil fertility levels, with the most prominent increment in TK (119.41%), highlighting their unique capacity to mobilize insoluble potassium in soil.

Concurrent with the improvement of soil nutrient availability, microbial agents exerted a significant suppressive effect on cotton *Fusarium* and *Verticillium* wilt under field conditions (**Table 1**). In Plot 1, CK1 exhibited a disease incidence of 28% and a disease index of 7.75; S1 reduced the incidence and disease index to 10% and 2.5, respectively, achieving a disease control efficacy of 67.74%. C1 further ameliorated disease symptoms, with the incidence and disease index reduced to 6% and 1.5, corresponding to a control efficacy of 80.65%. In Plot 2, CK2 showed a disease incidence of 21% and a disease index of 5.25; S2 lowered the incidence to 8% and the disease index to 2 (control efficacy: 61.90%), while C2 also reduced the incidence to 6% and the disease index to 1.5 (control efficacy: 71.43%).

**Table 1:** Field control efficacy of microbial agents against cotton *Fusarium* and *Verticillium* wilt.

Treatments	Disease incidence(%)	Disease index	Disease control efficacy (%)
CK1	28	7.75	—
S1	10	2.5	67.74
C1	6	1.5	80.65
CK2	21	5.25	—
S2	8	2	61.90
C2	6	1.5	71.43

CK1 and CK2: control groups, S1 and S2: single microbial agent treatments, C1 and C2: compound microbial agent treatments.

Amplicon sequencing results further confirmed that microbial agent treatments altered the rhizosphere microbial community structure. After quality control and filtering, all samples had a microbial community coverage approaching 1, indicating comprehensive sequencing of the rhizosphere microbial community. Specifically, bacterial amplicon sequence variants (ASVs) were 2684 ± 143, and fungal ASVs were 717 ± 80 (**Table 2**); rarefaction curves further confirmed that sequencing depth met the requirements for subsequent diversity analysis (**Supplementary Figure 1**). For α-diversity indices (ACE, Chao1, ASV counts), microbial agent treatments (S and C) did not exert a significant effect on soil bacterial communities, but significantly increased soil fungal community richness compared with the CK group. Meanwhile, the fungal Shannon and Simpson indices decreased in S, C treatments, indicating that microbial agents enhanced the abundance of dominant fungal taxa while reducing community evenness (**Table 2**). Additionally, principal coordinate analysis (PCoA) based on the Bray–Curtis distance revealed significant differences in both bacterial and fungal community structures between the S, C treatment groups and the CK group (**Supplementary Figure 2**), confirming that microbial agents effectively reshaped the rhizosphere microbial community composition.

**Table 2:** Richness estimators and diversity indices of bacterial and fungal communities across treatments.

Panel	Treatments	ACE	Chao1	ASV	Shannon	Coverage (%)
Bacteria	CK1	2729 ± 124 a	2722 ± 133 a	2661 ± 96 a	9.90 ± 0.123 a	82.7
	S1	2648 ± 76 a	2628 ± 73 a	2582 ± 62 a	9.94 ± 0.033 a	83.2
	C1	2590 ± 242 a	2578 ± 230 a	2550 ± 212 a	9.93 ± 0.074 a	83.4
	CK2	3020 ± 394 a	3010 ± 397 a	2954 ± 362 a	10.29 ± 0.003 a	86.4
	S2	2753 ± 170 a	2752 ± 184 a	2695 ± 150 a	10.294 ± 0.072 a	86.0
	C2	2708 ± 70 a	2692 ± 69 a	2665 ± 70 a	10.318 ± 0.045 a	86.4
Fungi	CK1	688 ± 63 b	698 ± 66 b	667 ± 63 b	5.80 ± 0.244 a	59.6
	S1	739 ± 48 ab	741 ± 52 ab	724 ± 43 ab	5.89 ± 0.453 a	57.6
	C1	885 ± 71 a	891 ± 77 a	852 ± 57 a	5.83 ± 0.176 a	55.2
	CK2	641 ± 18 b	646 ± 25 b	625 ± 14 b	6.19 ± 0.038 a	61.5
	S2	783 ± 56 a	788 ± 58 a	758 ± 54 a	6.22 ± 0.085 a	60.0
	C2	695 ± 39 ab	694 ± 39 ab	681 ± 32 ab	6.29 ± 0.028 a	61.7

Different lowercase letters indicate significant differences among treatments (*P < 0.05*).

**Figure 4:**
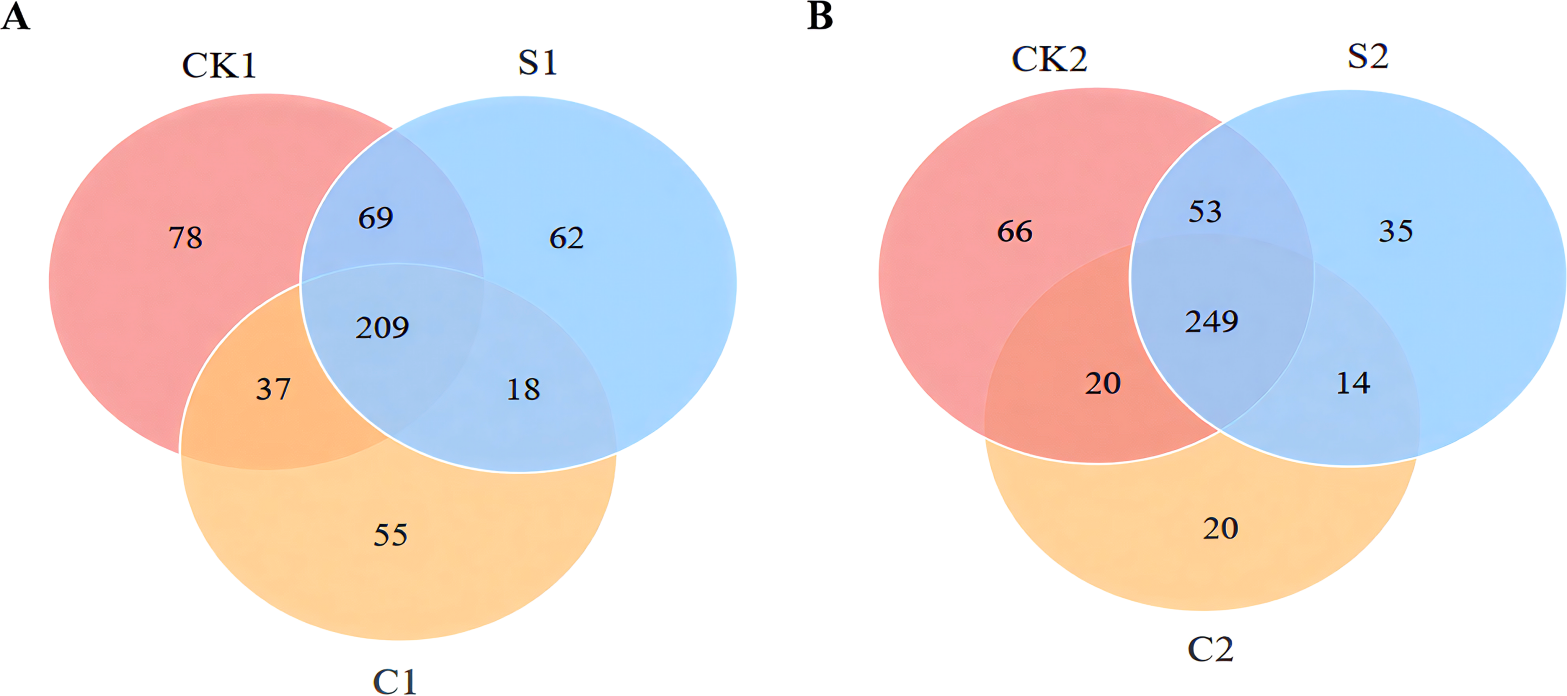
Venn diagram of bacterial genera counts in soil samples. **(A)** Plot 1; **(B)** Plot 2.

Analysis of bacterial communities in cotton rhizosphere soil showed that S and C microbial agents significantly reshaped bacterial genus distribution (**Figure 4**; **Supplementary Table 1**). In Plot 1, 209 bacterial genera were common to all groups, with 62, 55, and 78 unique genera in S1, C1, and CK1, respectively. Plot 2 showed a similar trend: 249 common genera were detected, with 35, 20, and 66 unique genera in S2, C2, and CK2, respectively. Further analysis showed S and C increased relative abundances of *Iamia*, *Polycyclovorans*, *Arenimonas*, and *Sphingobium*, but decreased those of *Streptomyces*, *Sphingomonas*, *Ensifer*, and *Dongia*.

**Figure 5:**
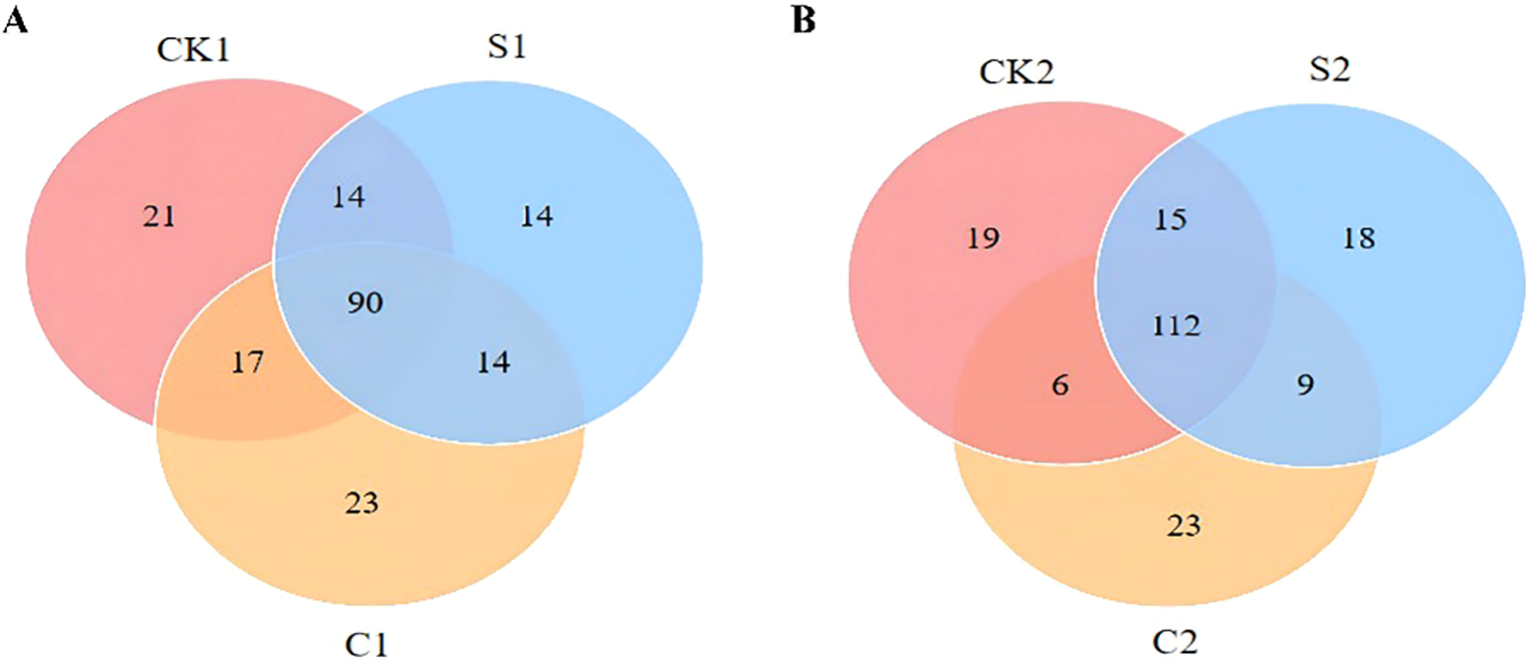
Venn diagram of fungal genera counts in soil samples. **(A)** Plot 1; **(B)** Plot 2.

For rhizosphere fungal communities, S and C microbial agents notably altered fungal genus distribution in comparison with CK (**Figure 5**; **Supplementary Table 2**). In Plot 1, 90 fungal genera were common across groups, with 14, 23, and 21 unique genera in S1, C1, and CK1, respectively. Plot 2 had 112 common genera, with 18, 23, and 19 unique genera in S2, C2, and CK2, respectively. S and C significantly increased relative abundances of *Penicillium*, *Metarhizium*, *Acrophialophora*, and *Talaromyces*, but decreased those of *Fusarium*, *Alternaria*, *Nectria*, and *Emericellopsis*.

### 3.3 Analysis of cotton rhizosphere soil secondary metabolites under microbial agent treatments

Untargeted metabolomics was employed to assay secondary metabolites in the cotton rhizosphere soil. Correlation analysis of quality control samples confirmed the reliability of identified differential metabolites (**Supplementary Figure 4**), laying a foundation for subsequent analysis of microbial-metabolite interactions. A total of 1655 metabolites were annotated via KEGG, belonging to 13 main categories including lipids and lipid-like molecules (28.79%), phenylpropanoids and polyketides (16.60%), and organic acids and derivatives (12.02%) (**Supplementary Figure 5**).

Microbial agents significantly reshaped the soil metabolite composition (**Supplementary Table 3**). The number of differential metabolites (up/down-regulated) varied among groups: 336 (192/144) in CK1.vs.S1 (e.g., up: Timosaponin A-III, 6-Chloropurine; down: Rehmannioside A, β-Asarone); 436 (293/143) in CK1.vs.C1 (e.g., up: SDMA, 1-Methylxanthine; down: Sinapyl Alcohol, Ergothioneine); 152 (118/34) in CK2.vs.S2 (e.g., up: Hydroxyecdysone, Polypodine B; down: Nonivamide, Alternariol); 243 (138/105) in CK2.vs.C2 (e.g., up: 15(R)-15-Methyl prostaglandin A2, Juglalin; down: Maltopentaose, Lignoceric Acid).

**Figure 6:**
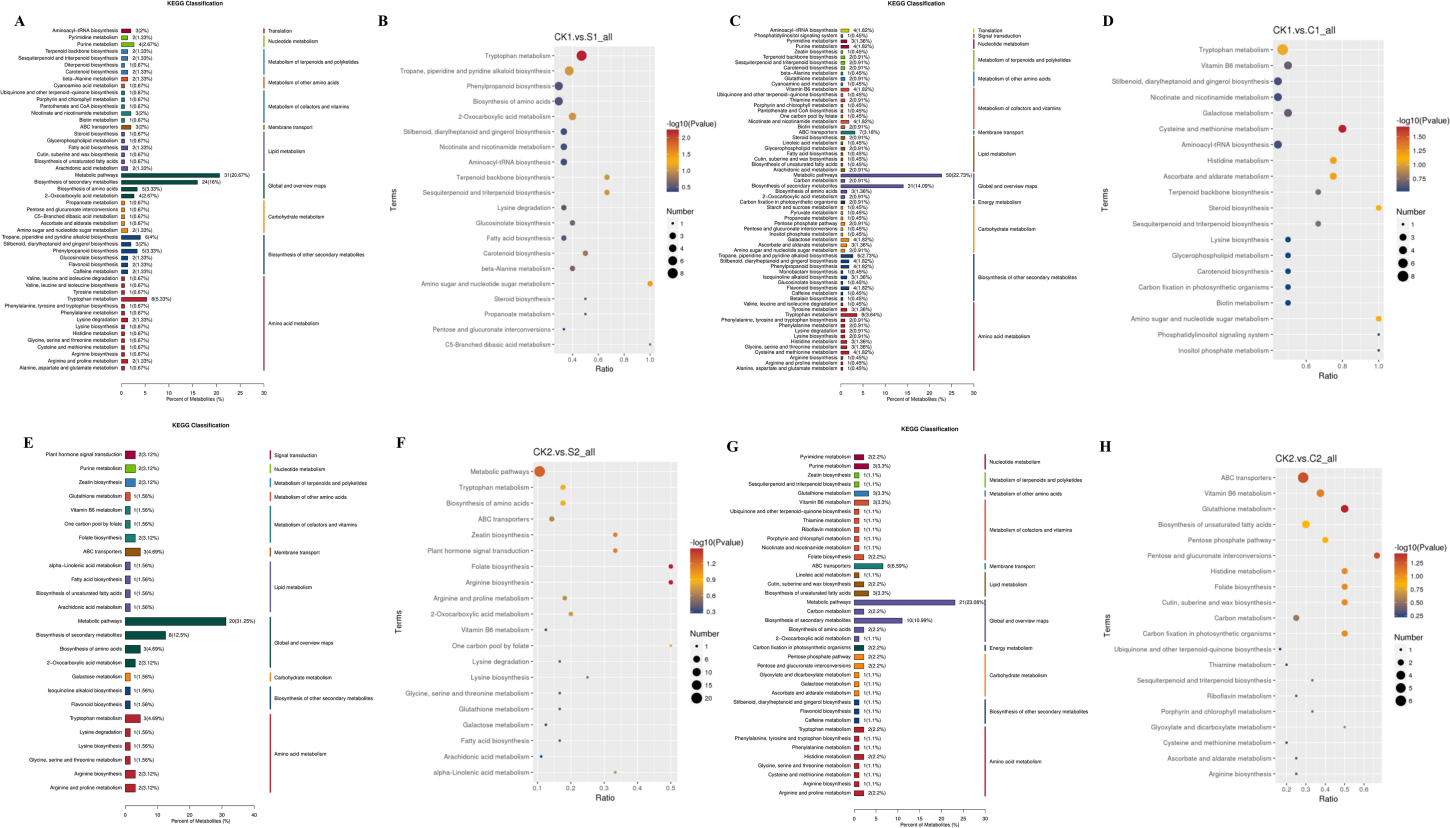
Differential metabolic pathway classification and KEGG enrichment analysis among treatment groups in cotton. **(A, B)** CK1.vs.S1; **(C, D)** CK1.vs.C1; **(E, F)** CK2.vs. S2; **(G, H)** CK2.vs.C2.

KEGG pathway enrichment analysis showed that these differential metabolites were involved in multiple pathways (**Figure 6**). Across CK1.vs.S1, CK1.vs.C1, CK2.vs.S2, and CK2.vs.C2 comparisons, these pathways shared consistent core characteristics, primarily encompassing amino acid metabolism, global/overview maps, secondary metabolite biosynthesis, lipid metabolism, and cofactor/vitamin metabolism (with group-specific variations). key pathways included tryptophan metabolism (consistently prominent), plus tropane/piperidine/pyridine alkaloid biosynthesis, amino acid biosynthesis, phenylpropanoid biosynthesis, purine metabolism (CK1.vs.S1); cysteine/methionine and tyrosine metabolism (CK1.vs.C1); arginine/proline metabolism and ABC transporters (CK2.vs.S2); and arginine/proline, histidine metabolism, and ABC transporters (CK2.vs.C2). Differentially accumulated metabolites (DAMs) enriched 22–38 KEGG pathways per comparison, with 20 significant ones each, mostly linked to lipids, phenylpropanoids, and polyketides.

**Figure 7:**
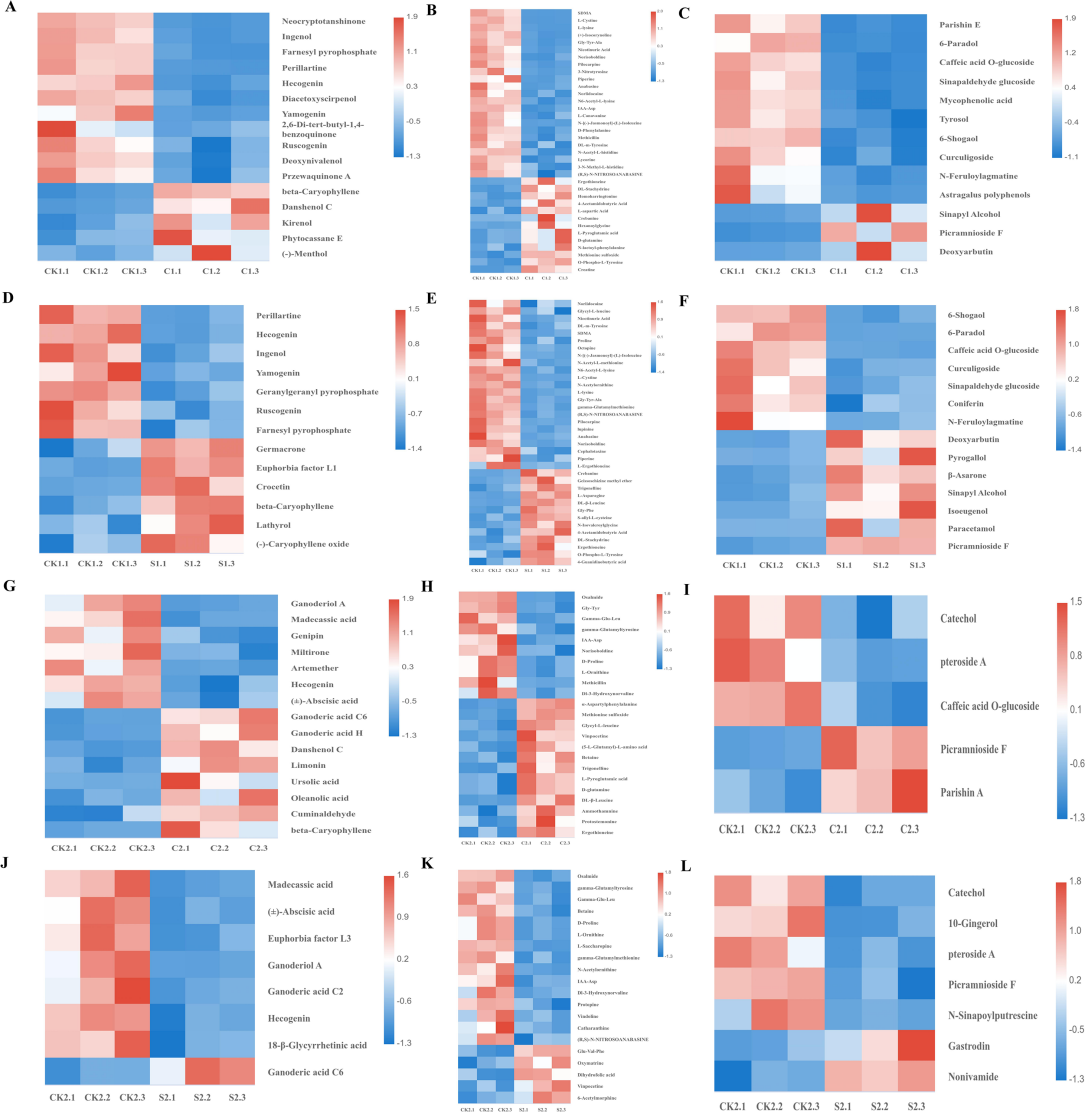
Heatmap of DAMs classification clustering. **(A–C)** CK1.vs.C1; **(D–F)** CK1.vs.S1; **(G–I)** CK2.vs. C2; **(J–L)** CK2.vs.S2, including terpenoids, nitrogen-containing compounds, and phenols.

Based on the above, we focused on three key categories of differentially accumulated metabolites (DAMs) from primary classification: terpenoids, nitrogen-containing compounds, and phenols. These metabolites critically shape the rhizosphere microbiome, mediate plant-microbe interactions, and regulate cotton growth and soil health (**Figure 7**). Specifically, CK1.vs.C1 included 16 terpenoids, 36 nitrogen-containing compounds, and 13 phenols as DAMs. CK1.vs.S1 included 13 terpenoids, 36 nitrogen-containing compounds, and 14 phenols as DAMs. CK2.vs.C2 included 15 terpenoids, 23 nitrogen-containing compounds, and 5 phenols as DAMs. CK2.vs.S2 included 8 terpenoids, 20 nitrogen-containing compounds, and 7 phenols as DAMs. Notably, in plot 1, key metabolites such as tryptamine, L-tryptophan, and serotonin were significantly increased in the microbial agent treatment compared with the control (**Supplementary Table 4**).

### 3.4 Effects of microbial agent treatments on cotton yield and fiber quality

Microbial agent applications exerted distinct impacts on the yield performance of cotton (**Table 3**). In Plot 1, the C1 treatment increased cottonseed yield by 20.17% and lint yield by 19.79% relative to the CK1. In Plot 2, the C2 further raised cottonseed yield and lint yield by 24.37% and 24.31%, respectively, compared to CK2. The S also exhibited a positive effect, increasing cottonseed and lint yields by 16.82% and 15.02% in Plot 1, and by 15.67% and 15.79% in Plot 2, respectively, though these increments were consistently lower than those achieved with the compound agent. These results collectively demonstrate that the compound microbial agent was consistently superior to the single agent in boosting both cottonseed and lint yield across the two experimental plots.

**Table 3:** Cotton yield and its components under different treatments.

Treatments	Cottonseed yield (kg/hm2)	Relative increase in cottonseed yield (%)	Lint cotton yield (kg/hm2)	Relative increase in lint cotton yield (%)	Lint percentage (%)
CK1	3062.92 ± 54.86c	—	2504.20 ± 37.61c	—	44.98
S1	3578.28 ± 55.87b	16.82	2880.38 ± 40.56b	15.02	44.60
C1	3684.82 ± 60.10a	20.17	2999.72 ± 48.55a	19.79	44.90
CK2	2108.68 ± 31.94c	—	1576.96 ± 25.74c	—	42.79
S2	2439.16 ± 39.51b	15.67	1825.90 ± 27.96b	15.79	42.81
C2	2622.48 ± 42.07a	24.37	1960.25 ± 31.96a	24.31	42.77

CK1 and CK2: control groups, S1 and S2: single microbial agent treatments, C1 and C2: compound microbial agent treatments. Different lowercase letters indicate significant differences among treatments (*P < 0.05*).

Alongside yield increases, compound agent C significantly improved cotton fiber quality (**Table 4**). Compared to CK, compound agent significantly increased fiber strength by 7.48% and spinning consistency index by 7.35%, while stabilizing micronaire at 4.6–5.0 (optimal range). In comparison with the S, C increased fiber strength by 4.86% and spinning consistency index by 8.80% and reduced fiber yellowness. This confirms compound agent can synergistically increase yield and improve fiber quality via microbial synergy.

**Table 4:** Effects of different treatments on cotton fiber quality.

Treatments	Upper half mean length (mm)	Uniformity index (%)	Fibre strength (cN·tex^-1^)	Micronaire	Elongation (%)	Reflectance (%)	Yellowness	Spinning consistency index
CK1	29.76 ± 0.92a	85.06 ± 1.19a	28.46 ± 0.70a	4.73 ± 0.57a	5.56 ± 0.64a	79.93 ± 1.20a	7.96 ± 0.05a	141.33 ± 7.63a
S1	29.26 ± 0.40a	83.93 ± 1.68a	29.36 ± 1.50a	4.7 ± 0.20a	5.2 ± 0.10a	80.40 ± 0.75a	7.5 ± 0.10b	137.66 ± 6.42a
C1	29.93 ± 0.40a	85.73 ± 0.61a	30.26 ± 2.55a	4.6 ± 0.00a	5.7 ± 0.20a	79.20 ± 0.79a	7.83 ± 0.30ab	150.66 ± 10.69a
CK2	28.93 ± 0.32a	83.8 ± 0.30a	27.76 ± 0.35a	5.10 ± 0.10a	4.73 ± 0.11ab	78.40 ± 1.08a	7.83 ± 1.52a	127.33 ± 1.52a
S2	28.06 ± 0.55b	83.53 ± 0.58a	28.26 ± 0.40a	4.83 ± 0.57a	4.60 ± 0.10b	79.10 ± 1.05a	7.96 ± 0.57a	127.33 ± 4.50a
C2	28.96 ± 0.25a	84.36 ± 0.47a	30.16 ± 2.44a	5.00 ± 0.20a	4.90 ± 0.17a	78.66 ± 0.80a	7.60 ± 0.10b	137.66 ± 11.01a

CK1 and CK2: control groups, S1 and S2: single microbial agents, C1 and C2: compound microbial agents. Different lowercase letters indicate significant differences among treatments (*P < 0.05*).

### 3.5 Correlation analysis of key indicators under microbial agent treatments

**Figure 8:**
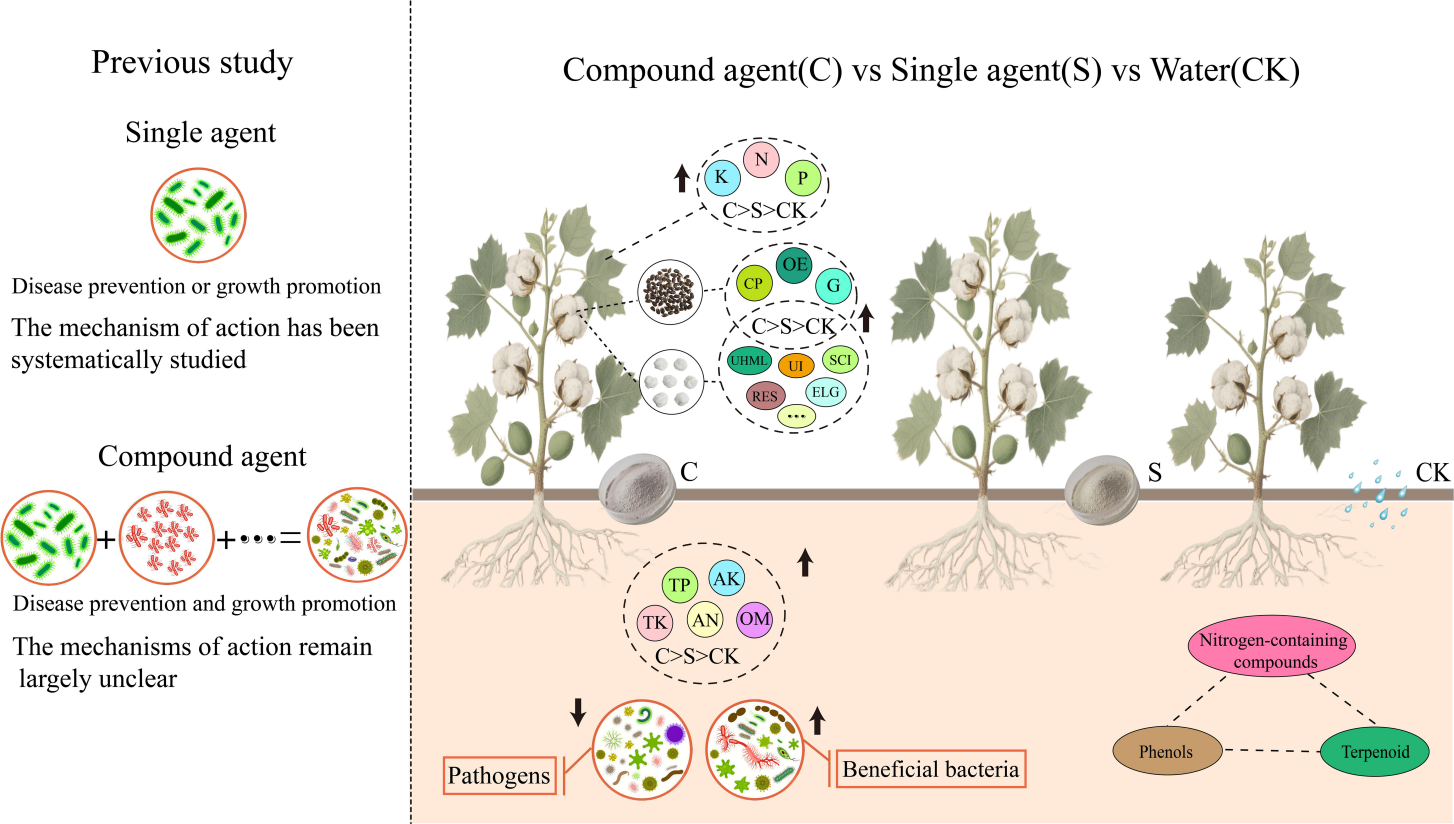
The Impact of microbial agents on cotton. This network illustrates the significant correlations among microbial agent treatments (C, S), cotton physiological indices, soil nutrients, rhizosphere microbial genera, secondary metabolites, and yield and fiber quality traits.

Multi-dimensional Spearman correlation analysis was performed to clarify the relationships among microbial agent treatments, cotton physiological traits, soil nutrients, rhizosphere microbial communities, secondary metabolites, yield, and fiber quality (**Figure 8**). The synthetic compound microbial agent (C) showed significant positive correlations with cotton leaf chlorophyll content, antioxidant enzyme activities (CAT, POD, SOD), root activity, soil available phosphorus (AP), total potassium (TK), and organic matter (OM), and also exhibited strong negative correlations with leaf malondialdehyde (MDA) content and *Fusarium* wilt and *Verticillium* wilt disease index, indicating that C can simultaneously enhance plant stress resistance and improve soil fertility. Beneficial rhizosphere microbes induced by C were positively correlated with soil AP, TK, and cotton single boll weight, while negatively correlated with pathogenic genera (e.g., *Fusarium*, *Alternaria*), confirming that C reshapes the rhizosphere microbial community to create a favorable microecological environment for cotton growth. Key differential metabolites regulated by C (e.g., terpenoids, nitrogen-containing compounds, phenols) were positively correlated with cotton leaf phosphorus content and spinning consistency index, indicating that metabolic pathway modulation by C contributes to improved nutrient absorption and fiber quality. These results collectively demonstrate that compound microbial agents drive synergistic and coordinated improvements in cotton growth, disease resistance, yield, and fiber quality through three interconnected regulatory pathways: activating soil insoluble nutrients (especially phosphorus and potassium); reshaping rhizosphere microbial communities to enrich beneficial taxa and inhibit pathogens; modulating rhizosphere metabolic profiles to accumulate functional metabolites. This multi-dimensional regulatory mechanism endows compound agents with significant synergistic advantages over single agents.

## 4 Discussion

### 4.1 Performance of compound microbial agent in promoting cotton yield and fiber quality

This study was conducted to meet the requirements of national food security strategies and agricultural green development. To reduce reliance on chemical pesticides and fertilizers and promote the green transformation of the cotton industry, we synthesized a compound microbial agent tailored to the ecological conditions of Xinjiang cotton-growing areas. Evaluation results from two experimental plots in 2024 showed that application of this agent significantly increased cotton yield by 20.00–24.34% and improved fiber quality. Specifically, the spinning consistency index, breaking fiber strength, and elongation at break were increased by 7.35%, 7.48%, and 3.06%, respectively. To verify the consistency and stability of its efficacy, an additional replicated experiment was conducted in expanded experimental plots in 2025. The results further confirmed that this agent exhibited a reliable yield-increasing effect, with a yield increase of 22.49%, confirming its stable performance in enhancing cotton production (**Supplementary Table 5**).

The findings of this study are consistent with existing research on the growth-promoting mechanisms of microorganisms. For example, Ren et al. demonstrated that applying *Bacillus subtilis* alleviated drought stress in cotton, increasing yield by 8.94–9.28%, a finding consistent with our results (Ren et al., 2025). Complementing these yield-related observations, the work of El-Waraky, Li, Xie et al. has also validated the efficacy of microbial agent in optimizing cotton fiber quality, with key fiber quality indicators showing marked improvements after treatment with tailored microbial agents (El-Waraky et al., 2025; Li et al., 2025; Xie et al., 2025). Similarly, compound microbial agents typically achieve synergistic improvements in crop yield and quality through multiple pathways, including promoting root development, enhancing nutrient uptake, and regulating the rhizosphere microbial community (Zhang et al., 2025a; Deng et al., 2021; Zhao et al., 2024). Furthermore, our findings confirmed that synergistic interactions between microorganisms can effectively enhance the systemic stress resistance of plants—a conclusion also supported by previous studies (Niu et al., 2017; Du et al., 2022; Fan et al., 2025). This provides a theoretical basis for explaining why the compound microbial agent exhibited superior efficacy over single-strain preparations in the present study. Pot experiments were further conducted to compare the effects of the independently synthetic agent and commercially available microbial agents on the growth and physiological characteristics of cotton seedlings. Our results demonstrated that the synthetic microbial agent exerted superior effects on plant biomass accumulation, chlorophyll synthesis, and stress resistance, which aligns well with the findings reported by Yang et al. and thus highlights its great potential in promoting plant growth and enhancing stress tolerance (Yang et al., 2023). Collectively, these findings not only provide a feasible microbial technology-based approach for reducing the application of chemical fertilizers and pesticides and realizing green production in cotton-growing areas of Southern Xinjiang but also lay a theoretical and practical foundation for the large-scale popularization of this agent.

### 4.2 Rhizosphere microecological regulation and disease control efficacy of compound microbial agent

Building on the experimental observation that cotton yield was significantly increased, this study further investigated changes in the structure of the rhizosphere soil microbial community. Soil microbial community diversity is generally recognized as a crucial indicator for regulating soil ecosystems (Singh et al., 2024). Previous studies have shown that microbial agents can directly influence the composition of the rhizosphere microbial community via their intrinsic properties and indirectly regulate its diversity and structure by improving soil physicochemical properties (Aurodeepa et al., 2024; Philippot et al., 2024). The results of this study showed that, compared with the control group, application of the compound microbial agent reduced the Chao1 index and increased the Shannon index of the soil bacterial community, while increasing both the Chao1 and Shannon indices of the fungal community. Although *Bacillus* species were the main components of the tested agent, the relative abundance of *Bacillus* in the rhizosphere soil was significantly reduced by 35.37% following inoculation. This phenomenon may be attributed to competition, antagonism, or synergistic interactions between the microorganisms introduced by the agent and indigenous soil microbes (Li et al., 2025); alternatively, it may result from the agent’s indirect effects on the abundance of specific microbial taxa by regulating the rhizosphere environment (Krishnan et al., 2025). This change further indicates that the crop growth-promoting effect of the agent may not only rely on the direct colonization of its contained strains, but more closely associate with its comprehensive regulation of the rhizosphere microecosystem.

Intricate interrelationships exist among *Bacillu*s, *Fusarium oxysporum* and *Verticillium dahliae* in the rhizosphere microecosystem (Hasan et al., 2020). As common beneficial rhizosphere microbes, *Bacillus* can inhibit the growth and infection of these pathogens via multiple mechanisms, including ecological niche competition, secretion of antimicrobial substances, and induction of systemic resistance in plants (Lastochkina et al., 2019). The results showed that following application of the compound microbial agent, the relative abundance of *Verticillium* in the rhizosphere soil increased, while that of *Fusarium* decreased by 21.00%. This phenomenon indicates that the disease control effect of the agent may not directly manifest as the synchronous reduction in the abundance of all pathogenic taxa; instead, it is achieved through a combination of pathways, including regulating microbial community structure, altering interspecific interaction patterns, and enhancing plant resistance (Hajji-Hedfi et al., 2025). Subsequent field trials on disease control efficacy demonstrated that the compound agent achieved an overall control effect of 71.43–80.65% against cotton *Fusarium* wilt and *Verticillium* wilt. These results confirmed its capacity to effectively suppress disease progression while modulating the population dynamics of pathogenic microbes. Notably, the rhizosphere microecological environment is one of the most complex ecosystems in soil (Balla et al., 2021). The long-term effects of agent application on microbial networks, as well as the causal relationships between these effects and crop health, remain to be further verified. These findings also provide an important theoretical basis and research directions for the synthesis and optimization of compound microbial agents in future research.

### 4.3 Regulation of compound microbial agent on secondary metabolites in cotton rhizosphere soil

Although research on the effects of microbial agents on secondary metabolites in cotton rhizosphere soil remains limited, this study used untargeted metabolomics to investigate the impacts of compound microbial agent application on secondary metabolites in cotton rhizosphere soil. The results showed that the differential metabolites induced by the agent treatment were mainly enriched in metabolic pathways, including lipids and lipid-like molecules, organoheterocyclic compounds, phenylpropanoids and polyketides, and organic acids and their derivatives. These changes primarily involved three classes of substances: terpenoids, nitrogen-containing compounds, and phenols. Notably, the agent treatment significantly enhanced the accumulation of tryptamine, L-tryptophan, and serotonin (Bacler-Żbikowska et al., 2025). Among these, tryptamine not only acts as a precursor for the synthesis of auxin and melatonin, but also plays a crucial role in signal transduction during plant-microbe interactions (Wang et al., 2025). Previous studies have shown that volatile organic compounds (VOCs) released by rhizosphere microbes can affect the distribution and transport of auxin by regulating the expression of auxin-related genes (e.g., the auxin efflux carrier gene *At2g17500*) and altering flavonoid metabolism, thereby promoting plant root development and systemic resistance (Hao et al., 2016). Auxin can regulate plant growth and development and enhance resistance to pathogen infection (Caruana et al., 2020; Ratnaningsih et al., 2023; Bao et al., 2024); melatonin, as a pleiotropic phytohormone, exerts multiple physiological functions, including antioxidant activity, alleviation of nitrosative stress, maintenance of photosynthetic efficiency, and regulation of stress responses (Wang et al., 2024; Khan et al., 2025; Wu et al., 2025). Collectively, these metabolite alterations indicate that the agent treatment indirectly promotes cotton growth and enhances stress tolerance by regulating the rhizosphere metabolic environment.

### 4.4 Research priorities and industrialization prospects for the compound agent

The compound microbial agent outperforms single-strain counterparts in promoting cotton growth, enhancing stress tolerance, and improving yield and fiber quality. This superiority arises from the synergism of strains with distinct origins, biological traits, and modes of action, which mitigates key limitations of single-strain agents, elevates microbial biocontrol efficacy, and extends the persistence of biocontrol activity (Nunes et al., 2024; Hu et al., 2024). For example, the combination of multiple microbes is more stable across a wider variety of ecological and environmental conditions (Qian et al., 2020; Trivedi et al., 2020; Tabacchioni et al., 2021). Similar findings in alfalfa salt-alkali stress studies demonstrated that the compound agent more effectively enhanced plant stress resistance and soil physicochemical properties than the single-strain agent, confirming the cross-crop universality of its synergistic effect (Han et al., 2025).

Future research will focus on mechanism refinement, efficacy validation, and technological translation, advancing three core objectives: first, deciphering the molecular mechanisms of inter-strain synergy, optimizing formulation ratios and fermentation processes to enhance efficacy and stability; second, conducting 3 to 5-year long-term field trials across multiple cotton-growing regions to evaluate the agent’s long-term ecological safety and mitigation effect on continuous cropping obstacles, while refining supporting application technologies; third, promoting pilot-scale production and industrial adaptation, establishing standardized protocols for integrated application with drip irrigation systems and reduced chemical fertilizers. This work is expected to clarify the paradigm of rhizosphere microecological synergistic regulation, facilitate large-scale deployment of the agent in Xinjiang cotton regions, achieve the dual benefit of reducing chemical inputs and improving cotton yield and quality, provide technical support for the green transition of cotton production in arid areas, and offer theoretical insights for developing compound microbial agents for analogous crops.

## 5 Conclusion

This study systematically demonstrates that a novel synthetic *Bacillus* compound microbial agent significantly enhances cotton productivity by increasing yield within a range of 20.00%–24.34% while concurrently improving critical fiber quality traits, notably spinning consistency index and breaking strength. These effects are achieved through a distinct “microbe–metabolite–plant” synergistic regulatory network. The agent facilitates nutrient mobilization, particularly phosphorus and potassium, ameliorates rhizosphere soil physicochemical characteristics, and restructures the resident microbial community via selective enrichment of plant-beneficial genera alongside suppression of pathogenic taxa. Additionally, it redirects secondary metabolism in cotton roots, elevating the accumulation of terpenoids and nitrogen-containing compounds, with marked enrichment of bioactive metabolites such as tryptamine and L-tryptophan. Relative to single-strain formulations, the compound agent exhibits distinctly superior synergy, as evidenced by significantly elevated antioxidant enzyme activities, enhanced chlorophyll content, improved root vitality, attenuated membrane lipid peroxidation, and overall strengthened stress resilience in cotton seedlings. This research offers an effective biological strategy to mitigate continuous cropping obstacles in Xinjiang cotton production, enhance yield stability, and decrease dependence on chemical fertilizers and pesticides, thereby supporting the green production of cotton in arid regions and informing the development and application of compound microbial agents.

## Data Availability

The data presented in the study are deposited in the MetaboLights repository, accession number MTBLS13947.
