# Supplementary material for: A synthetic Bacillus compound agent enhances cotton yield and fiber quality by regulating rhizosphere microbes and metabolites
Source: Front Plant Sci. 2026 Mar 9;17:1774588. doi: 10.3389/fpls.2026.1774588 (PMC13007723; doi:10.3389/fpls.2026.1774588)
Supplement: Supplementary file 1 [file DataSheet1.docx]

Supplementary Material

# Supplementary Figures and Tables

## Supplementary Figures

| 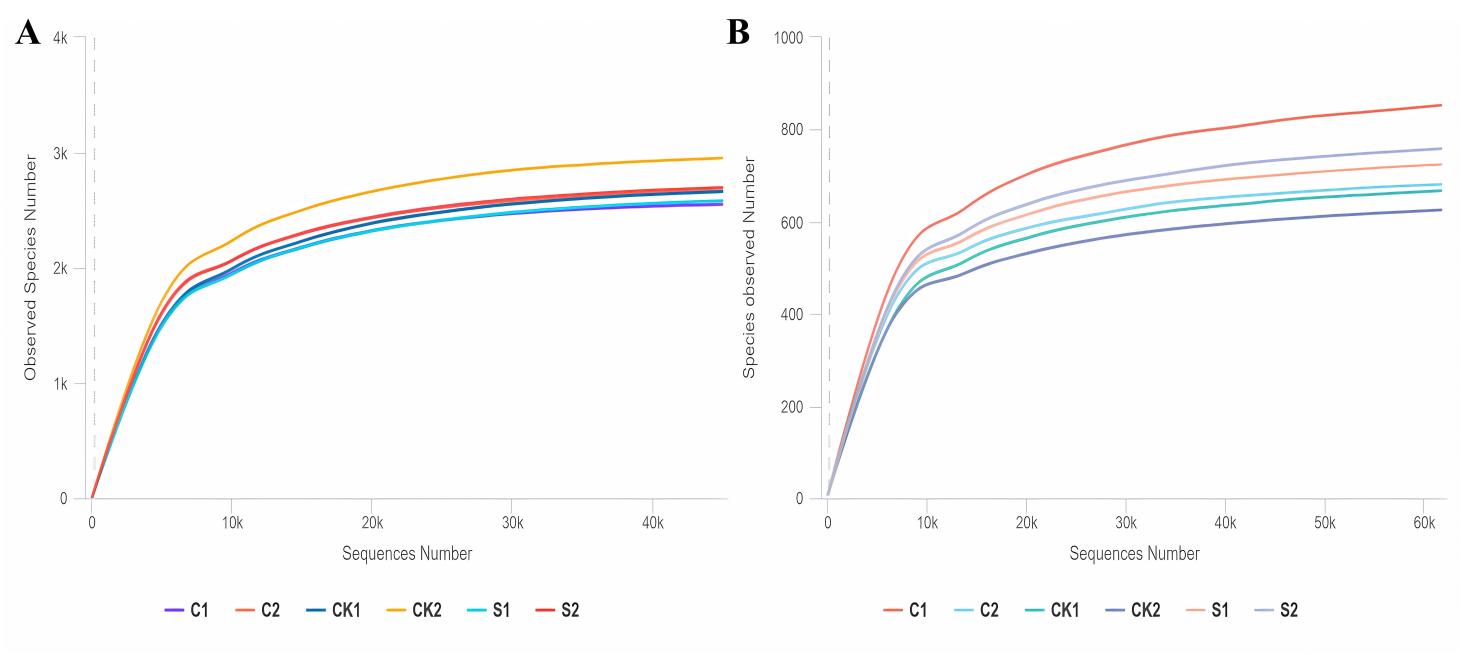**Supplementary Figure S1.** Rarefaction curves of microbial samples in cotton rhizosphere soil under different treatments. (A) bacterial, (B)fungal.   \| 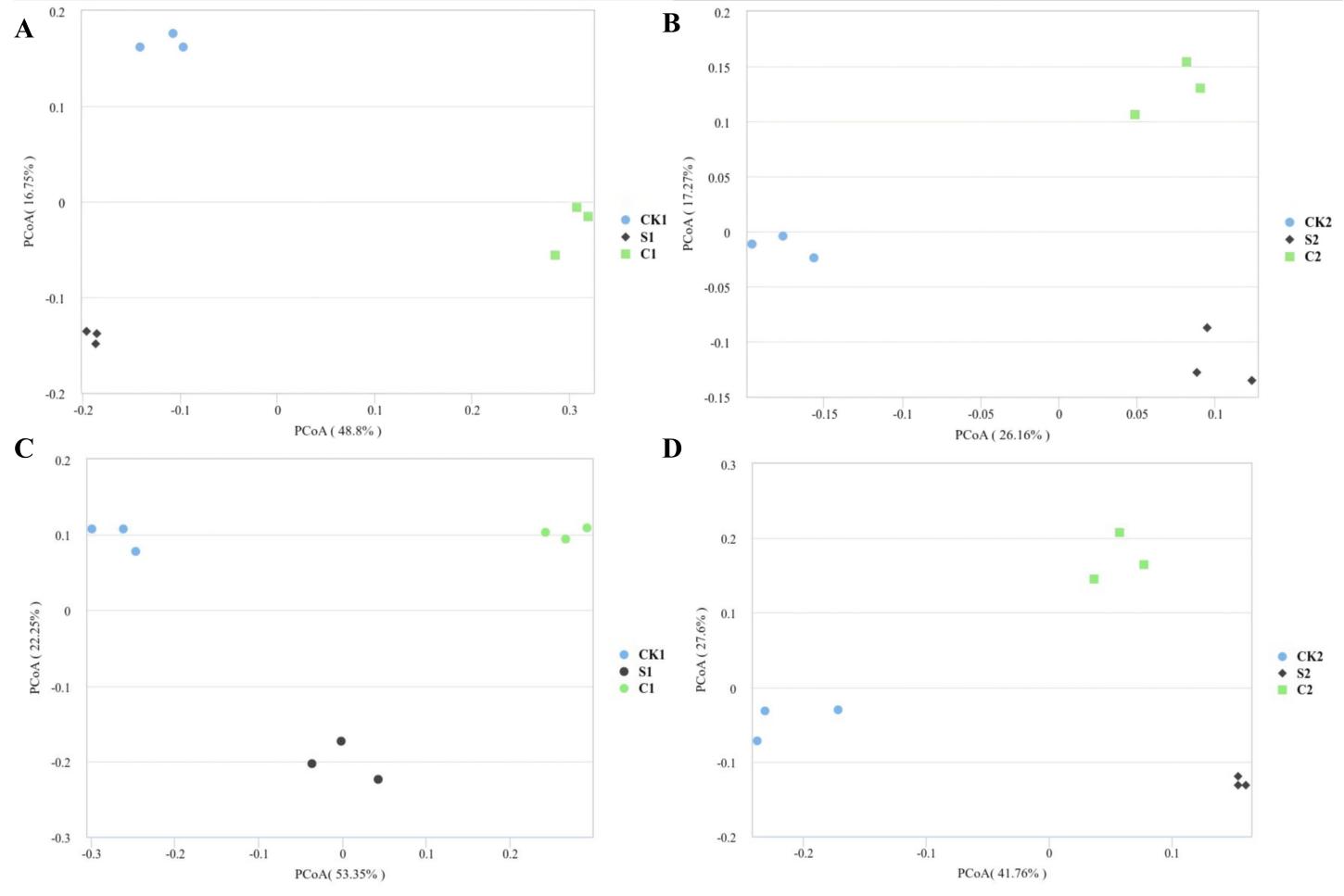 \| \| --- \| |
| --- | --- |
| **Supplementary Figure S2.** Principal component analysis of ASVs reveals soil microbial community structures. Bacterial: (A)plot 1, (B)plot 2; Fungal: (C) plot 1, (D)plot 2.   \| 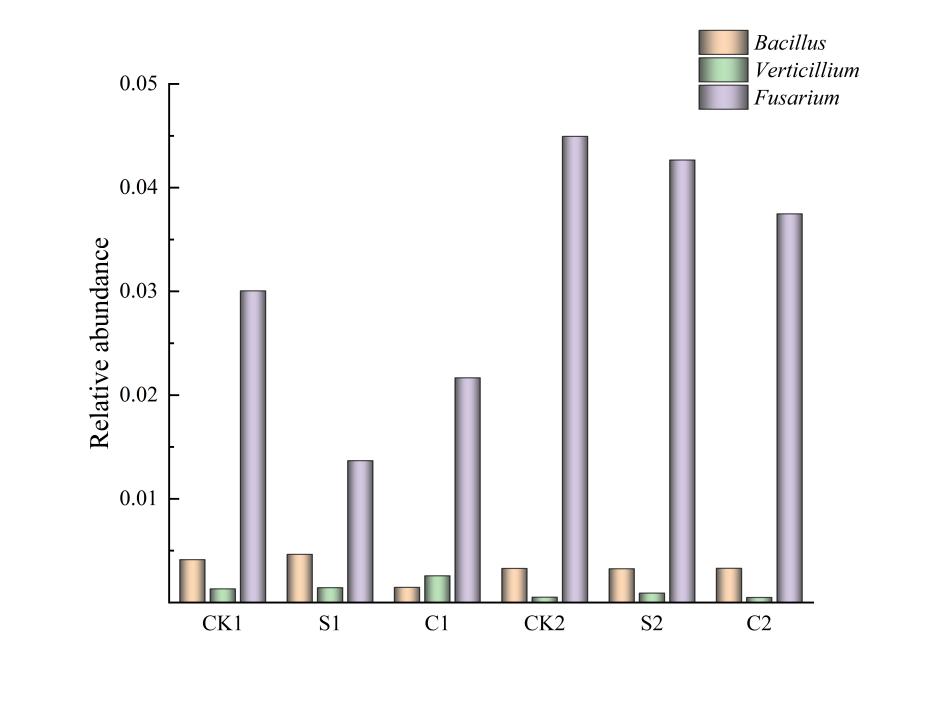 \| \| --- \|   **Supplementary Figure S3.** Effect of microbial agents on the abundance of soil microorganisms.   \| 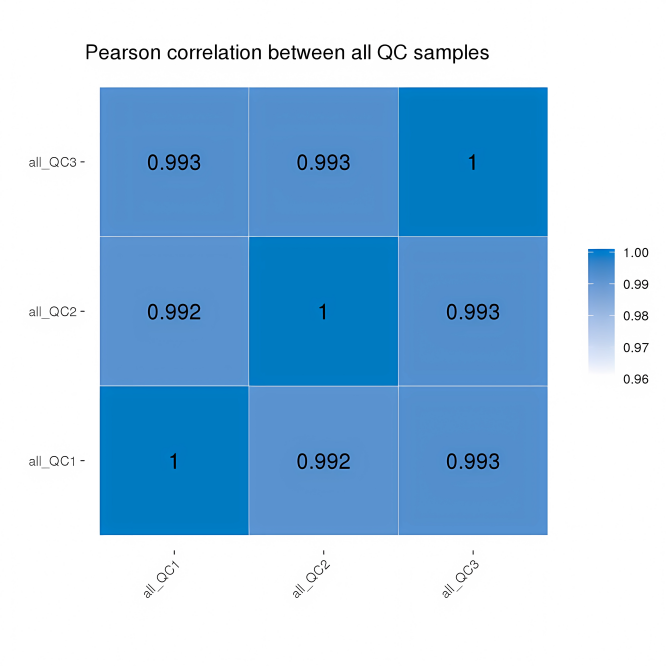 \| \| --- \|   **Supplementary Figure S4.** Group correlation plot. |
| \| 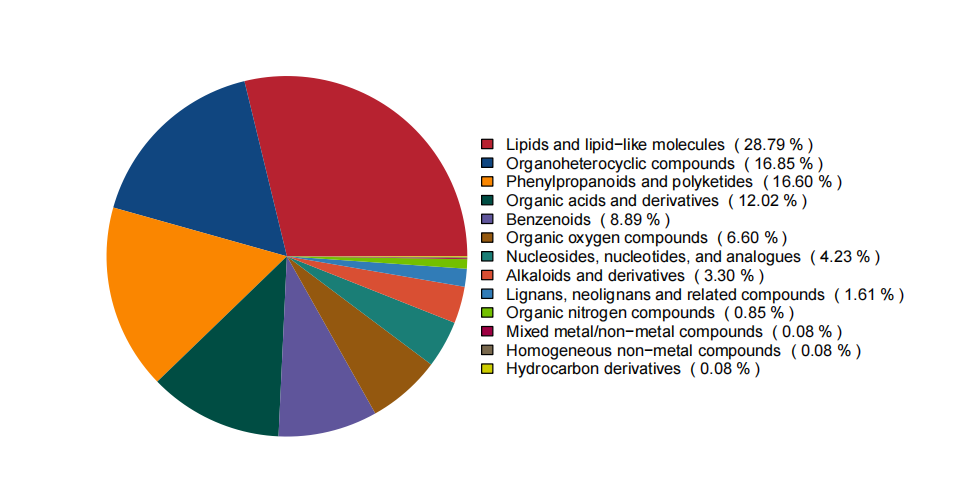 \| \| --- \| |

**Supplementary Figure S5.** Effect of microbial agents on the abundance of soil microorganisms Metabolite classification pie chart.

## Supplementary Tables

**Supplementary Tables S1.** Relative abundances of bacterial genera in soil samples.

| Taxonomy | Samples | | | | | |
| --- | --- | --- | --- | --- | --- | --- |
| Genera (phyla) | CK1 | S1 | C1 | CK2 | S2 | C2 |
| *Iamia* | 0.551% | 0.617% | 0.741% | 0.806% | 0.953% | 1.206% |
| *Polycyclovorans* | 0.239% | 0.242% | 0.266% | 0.018% | 0.080% | 0.018% |
| *Arenimonas* | 0.211% | 0.315% | 0.274% | 0.096% | 0.106% | 0.129% |
| *Sphingobium* | 0.104% | 0.193% | 0.265% | 0.016% | 0.032% | 0.016% |
| *Nitrospira* | 0.262% | 0.338% | 0.640% | 0.178% | 0.188% | 0.183% |
| *Paenibacillus* | 0.057% | 0.068% | 0.076% | 0.057% | 0.068% | 0.076% |
| *Sandaracinus* | 0.027% | 0.030% | 0.028% | 0.028% | 0.033% | 0.041% |
| *Sumerlaea* | 0.048% | 0.048% | 0.073% | 0.051% | 0.068% | 0.076% |
| *Hassallia* | 0.033% | 0.046% | 0.042% | 0.004% | 0.006% | 0.008% |
| *Tagaea* | 0.080% | 0.096% | 0.113% | 0.049% | 0.065% | 0.065% |
| *Streptomyces* | 0.567% | 0.502% | 0.306% | 1.727% | 1.564% | 1.473% |
| *Sphingomonas* | 1.550% | 1.184% | 1.238% | 3.847% | 2.150% | 2.362% |
| *Ensifer* | 0.670% | 0.381% | 0.259% | 0.556% | 0.209% | 0.259% |
| *Dongia* | 0.538% | 0.348% | 0.448% | 0.759% | 0.698% | 0.680% |
| *Solirubrobacter* | 0.097% | 0.048% | 0.061% | 0.630% | 0.397% | 0.580% |
| *Kocuria* | 0.047% | 0.000% | 0.000% | 0.574% | 0.329% | 0.210% |
| *Lysobacter* | 0.695% | 0.680% | 0.426% | 0.399% | 0.303% | 0.316% |
| *Fluviicola* | 0.018% | 0.002% | 0.010% | 0.003% | 0.002% | 0.002% |
| *Nocardioides* | 0.200% | 0.083% | 0.086% | 1.182% | 0.330% | 0.527% |
| *Ilumatobacter* | 0.323% | 0.548% | 0.381% | 0.341% | 0.237% | 0.279% |
| *Mariniflexile* | 0.177% | 0.250% | 0.031% | 0.024% | 0.007% | 0.004% |
| *Acidibacter* | 0.100% | 0.140% | 0.259% | 0.219% | 0.148% | 0.188% |
| *Candidatus Nitrosotenuis* | 0.134% | 0.469% | 0.568% | 0.144% | 0.142% | 0.112% |
| *Bacillus* | 0.413% | 0.464% | 0.146% | 0.325% | 0.332% | 0.331% |

CK1 and CK2: control groups, S1 and S2: single microbial agents, C1 and C2: compound microbial agents.

**Supplementary Tables S2.** Relative abundances of fungal genera in soil samples.

| Taxonomy | Samples | | | | | |
| --- | --- | --- | --- | --- | --- | --- |
| Genera (phyla) | CK1 | S1 | C1 | CK2 | S2 | C2 |
| *Penicillium* | 0.188% | 0.676% | 0.364% | 0.389% | 0.559% | 0.858% |
| *Metarhizium* | 0.105% | 0.134% | 0.548% | 0.081% | 0.243% | 0.524% |
| *Acrophialophora* | 0.009% | 0.019% | 0.026% | 0.048% | 0.159% | 0.080% |
| *Talaromyces* | 0.016% | 0.054% | 0.060% | 0.000% | 0.007% | 0.006% |
| *Ochroconis* | 0.038% | 0.054% | 0.051% | 0.060% | 0.121% | 0.109% |
| *Preussia* | 0.321% | 0.327% | 0.426% | 0.359% | 0.541% | 1.736% |
| *Cephalotrichum* | 0.924% | 3.066% | 1.178% | 0.626% | 1.240% | 1.232% |
| *Mortierella* | 0.812% | 2.401% | 1.645% | 0.133% | 0.282% | 0.381% |
| *Wardomyces* | 1.513% | 1.817% | 2.430% | 0.822% | 1.421% | 1.498% |
| *Fusarium* | 3.000% | 1.360% | 2.168% | 4.492% | 4.264% | 3.751% |
| *Alternaria* | 2.873% | 1.683% | 0.741% | 8.328% | 3.577% | 6.243% |
| *Nectria* | 0.391% | 0.189% | 0.354% | 0.391% | 0.189% | 0.354% |
| *Emericellopsis* | 0.319% | 0.121% | 0.030% | 0.637% | 0.410% | 0.176% |
| *Papulaspora* | 0.183% | 0.193% | 0.157% | 0.301% | 0.131% | 0.184% |
| *Spizellomyces* | 0.327% | 0.004% | 0.024% | 0.327% | 0.004% | 0.022% |
| *Filobasidium* | 2.031% | 1.196% | 0.335% | 3.982% | 2.143% | 3.068% |
| *Chaetomium* | 0.591% | 0.076% | 0.082% | 0.085% | 0.057% | 0.050% |
| *Papiliotrema* | 0.046% | 0.012% | 0.007% | 0.061% | 0.011% | 0.055% |
| *Pseudogymnoascus* | 0.594% | 0.396% | 0.147% | 0.454% | 0.603% | 0.757% |
| *Botryotrichum* | 14.717% | 4.361% | 2.302% | 1.293% | 5.062% | 2.993% |
| *Cladosporium* | 5.620% | 7.713% | 6.649% | 8.444% | 5.087% | 6.137% |
| *Geosmithia* | 0.089% | 0.303% | 1.379% | 0.097% | 0.062% | 0.037% |
| *Acaulium* | 0.637% | 0.423% | 0.292% | 0.588% | 0.983% | 1.384% |
| *Lecanicillium* | 0.063% | 0.281% | 0.001% | 0.194% | 0.060% | 0.049% |
| *Verticillium* | 0.131% | 0.143% | 0.257% | 0.051% | 0.090% | 0.048% |

CK1 and CK2: control groups, S1 and S2: single microbial agents, C1 and C2: compound microbial agents.

**Supplementary Tables S3.** Differential screening results of metabolites.

| Compared Samples | Total-number | Diff-number | Up-number | Down-number |
| --- | --- | --- | --- | --- |
| CK1.vs.S1 | 1655 | 336 | 192 | 144 |
| CK1.vs.C1 | 1655 | 436 | 293 | 143 |
| CK2.vs.S2 | 1655 | 152 | 118 | 34 |
| CK2.vs.C2 | 1655 | 243 | 138 | 105 |

**Supplementary Tables S4.** Abundance of selected metabolites.

| ID | Name | CK1.vs.C1_log_2_FC | CK1.vs.C1_P-value | CK1.vs.S1_log_2_FC | CK1.vs.S1_P-value |
| --- | --- | --- | --- | --- | --- |
| Com_31187_pos | Tryptamine | 1.537549841920819 | 0.0001130011800111 | 1.2215500484079291 | 0.0002669844035864 |
| Com_10115_pos | L-Tryptophan | 1.7301434172250665 | 0.0101581928871492 | 1.669376022316603 | 0.0011189958786703 |
| Com_12682_pos | Serotonin | 1.856377878586083 | 0.0156214382216636 | 2.1688608011820305 | 0.0103186415291275 |

**Supplementary Tables S5.** Cotton yield and its components under different treatments in the 2025 field experiment.

| Treatments | Cottonseed yield  (kg/hm2) | Relative increase in cottonseed yield（%） | Lint cotton yield  (kg/hm2) | Relative increase in lint cotton yield（%） | Lint percentage  （%） |
| --- | --- | --- | --- | --- | --- |
| CK | 3247.29 | — | 2249.97 | — | 40.92 |
| S | 3790.92 | 16.74 | 2626.44 | 16.73 | 40.92 |
| C | 3978.24 | 22.51 | 2755.09 | 22.45 | 40.91 |

CK: control groups, S: single microbial agent treatments, C1: compound microbial agent treatments.
